# Prevalence of Metabolic Syndrome and Its Components among Japanese Workers by Clustered Business Category

**DOI:** 10.1371/journal.pone.0153368

**Published:** 2016-04-15

**Authors:** Tomoo Hidaka, Takehito Hayakawa, Takeyasu Kakamu, Tomohiro Kumagai, Yuhei Hiruta, Junko Hata, Masayoshi Tsuji, Tetsuhito Fukushima

**Affiliations:** 1 Department of Hygiene and Preventive Medicine, School of Medicine, Fukushima Medical University, Fukushima City, Fukushima, Japan; 2 Fukushima Branch Office, Japan Health Insurance Association, Fukushima City, Fukushima, Japan; 3 Department of Preventive Medicine and Public Health, Faculty of Medicine, Fukuoka University, Fukuoka City, Fukuoka, Japan; Centers for Disease Control and Prevention, UNITED STATES

## Abstract

The present study was a cross-sectional study conducted to reveal the prevalence of metabolic syndrome and its components and describe the features of such prevalence among Japanese workers by clustered business category using big data. The data of approximately 120,000 workers were obtained from a national representative insurance organization, and the study analyzed the health checkup and questionnaire results according to the field of business of each subject. Abnormalities found during the checkups such as excessive waist circumference, hypertension or glucose intolerance, and metabolic syndrome, were recorded. All subjects were classified by business field into 18 categories based on The North American Industry Classification System. Based on the criteria of the Japanese Committee for the Diagnostic Criteria of Metabolic Syndrome, the standardized prevalence ratio (SPR) of metabolic syndrome and its components by business category was calculated, and the 95% confidence interval of the SPR was computed. Hierarchical cluster analysis was then performed based on the SPR of metabolic syndrome components, and the 18 business categories were classified into three clusters for both males and females. The following business categories were at significantly high risk of metabolic syndrome: among males, Construction, Transportation, Professional Services, and Cooperative Association; and among females, Health Care and Cooperative Association. The results of the cluster analysis indicated one cluster for each gender with a higher prevalence of metabolic syndrome components; among males, a cluster consisting of Manufacturing, Transportation, Finance, and Cooperative Association, and among females, a cluster consisting of Mining, Transportation, Finance, Accommodation, and Cooperative Association. These findings reveal that, when providing health guidance and support regarding metabolic syndrome, consideration must be given to its components and the variety of its prevalence rates by business category and gender.

## Introduction

Metabolic syndrome is a group of risk factors for cardiovascular disease and mortality that include central obesity, hypertension, glucose intolerance and dyslipidemia [1–3]. Furthermore, metabolic syndrome has, on a global scale, become one of the key challenges to the public health sector. Early studies reported that its prevalence was 20–30% in the U.S. [4,5], and recent Japanese studies have revealed a prevalence of 8–25% in male and 2–22% in female [6–8].

The prevalence of metabolic syndrome has recently been suggested to vary greatly depending on the subject’s business category; high prevalence of metabolic syndrome has been reported among the retired, unemployed [9], bus drivers [10], university employees [11], and workers in the agricultural industry [12] oil industry [13], and health care sector [14].

However, these studies did not compare the prevalence between, nor did they indicate the common features of prevalence in, different business categories. This was due to a lack of categories covered. Moreover, the classifications of business categories used in these past studies were inconsistent. It is required to conduct a study using standard classification of business category and analyze the prevalence of metabolic syndrome between all categories.

The North American Industry Classification System (NAICS) is a widely used standard classification system of business categories [15], and has been applied in various occupational health studies [16,17]. Therefore, NAICS was considered to be suitable for the present study. We hypothesized that when business categories are clustered, the features of metabolic syndrome and its components can be elucidated by business category. Clustering may also contribute to the identification of the common and/or distinctive features of metabolic syndrome, which can essentially aid in understanding the background of this disease and its components.

Here, we reveal for the first time the prevalence of metabolic syndrome and its components, and describe the features of such prevalence among Japanese workers by clustered business category using big data

## Methods

### Study Sample

In 2012, health checkups of 161,362 workers were conducted in Fukushima Prefecture, Japan, by the Japan Health Insurance Association (JHIA), a national representative organization of insurance for laborers. Individuals who were aged 34 years or younger or aged 76 years or older, who had been under the insurance system for less than one year, or whose information regarding diagnostic criteria was unavailable, were excluded from the study. The JHIA health checkup included a questionnaire asking the subject’s business category, and, together with the checkup data, was recorded in the JHIA database. Of the 161,362 subjects, those who underwent measurement of waist circumference, blood pressure, blood glucose, lipid, and metabolic syndrome were 120,100 (74.4%), 120,114 (74.4%), 120,090 (74.4%), 120,088 (74.4%), and 120,097 (74.4%), respectively.

### Measurements

#### Diagnostic criteria

According to the Japanese Committee for the Diagnostic Criteria of Metabolic Syndrome in 2005, metabolic syndrome is defined as an excessive waist circumference (≥85 cm in men and ≥90 cm in women) as well as the presence of one or more of the following symptoms; hypertension, glucose intolerance, and dyslipidemia [18,19]. Hypertension is defined by systolic blood pressure ≥130 mmHg, or diastolic blood pressure ≥85 mmHg, or the use of antihypertensive drugs. Glucose intolerance is defined by fasting glucose ≥110 mg/dL, or the use of drugs for diabetes. Dyslipidemia is defined by neutral fat ≥150 mg/dL, or HDL cholesterol <40 mg/dL, or the use of antihyperlipidemic drugs.

#### Business category

The business categories of the subjects were extracted from the JHIA database. The obtained information was classified based on the Japan Standard Industrial Classification, which is compatible with NAICS. Eighteen business categories were used in the present study: (1) Agriculture, (2) Mining, (3) Utilities, (4) Construction, (5) Manufacturing, (6) Wholesale Trade, (7) Transportation, (8) Information, (9) Finance, (10) Real Estate, (11) Professional Services, (12) Educational Services, (13) Health Care, (14) Arts, (15) Accommodation, (16) Cooperative Association, (17) Other Services, and (18) Public Administration. The details of the categories are shown in Table 1.

**Table 1:** Business Category Details.

Business categories used in this study	Proper name in NAICS	Examples of subclassification
**(1) Agriculture**	Agriculture, Forestry, Fishing and Hunting	Rice Farming, Logging
**(2) Mining**	Mining, Quarrying, and Oil and Gas Extraction	Iron Ore Mining, Stone Mining and Quarrying
**(3) Utilities**	Utilities	Electric Power Generation, Water Supply and Irrigation Systems
**(4) Construction**	Construction	Industrial Building Construction, Poured Concrete Foundation and Structure Contractors
**(5) Manufacturing**	Manufacturing	Seafood Product Preparation and Packaging, Industrial Machinery Manufacturing
**(6) Wholesale Trade**	Wholesale Trade and Retail Trade	Sporting Goods Stores, Automobile and Other Motor Vehicle Merchant Wholesalers
**(7) Transportation**	Transportation	Postal Service, Interurban and Rural Bus Transportation
**(8) Information**	Information	Software Publishers, Newspaper Publishers
**(9) Finance**	Finance and Insurance	Commercial Banking, Credit Unions
**(10) Real Estate**	Real Estate and Rental and Leasing	Real Estate Property Managers, Passenger Car Rental and Leasing
**(11) Professional Services**	Professional, Scientific, and Technical Services	Research and Development in the Physical, Engineering, and Life Sciences, Architectural Services
**(12) Educational Services**	Educational Services	Colleges, Universities, and Professional Schools, Computer Training
**(13) Health Care**	Health Care and Social Assistance	General Medical and Surgical Hospitals, Nursing Care Facilities
**(14) Arts**	Arts, Entertainment, and Recreation	Amusement and Theme Parks, Golf Courses and Country Clubs
**(15) Accommodation**	Accommodation and Food Services	Hotels, Restaurants and Other Eating Places
**(16) Cooperative Association**	Cooperative Association	Agricultural Cooperative, Post Office Savings Bank
**(17) Other Services**	Other Services (except Public Administration)	Waste Treatment and Disposal, Electronic and Precision Equipment Repair and Maintenance
**(18) Public Administration**	Public Administration	Executive Offices, Administration of Education Programs

### Statistical analyses

#### Age adjustment and calculation of standardized prevalence ratio with 95% CI

The subjects were classified into four age groups, and age adjustment was conducted by an indirect method based on the total subject population. The SPR was calculated as the ratio of observed prevalence to the expected prevalence for each business category. The expected prevalence for each business category was obtained by multiplying the number of people who fell into three specific categories (age group, business category, and abnormality) by the percentage of people who fell into the corresponding age group and abnormality categories of the total subject population. The 95% confidence interval (95% CI) of SPR was derived assuming a Poisson distribution for the observed numbers.

#### Hierarchical cluster analysis

Hierarchical cluster analysis based on agglomerative statistics using Ward’s method was conducted for the SPRs of metabolic syndrome components. The data were classified into three clusters of business categories for both males and females. The mean SPR of each metabolic syndrome component for each cluster was calculated, and the data were analyzed by SPSS statistics version 17.0.

### Ethics

This study was approved by the Ethics Committee of Fukushima Medical University (Application No. 1703).

## Results

The characteristics of the subjects are shown in Tables 2 and 3. Blood pressure abnormalities were most common in both males and females, at 53.9% and 34.9%, respectively. Approximately one-fifth of the male subjects had metabolic syndrome (22.2%); however, this was observed in very few females (4.4%). Of the business categories, (7) Transportation, (4) Construction, and (2) Mining showed the highest prevalences of metabolic syndrome at 25.7%, 21.0%, 20.5%, respectively, whereas (13) Health care, (18) Public Administration, (14) Arts, and (15) Accommodation showed the lowest prevalences at 8.7%, 11.4%, 12.1%, and 12.1%, respectively.

**Table 2:** Characteristics of Subjects.

		Waist circumference (n = 120100)		Blood pressure (n = 120114)		Blood glucose (n = 120090)		Lipid (n = 120088)		Metabolic syndrome (n = 120097)	
	Abnormalities	Observed	Not observed	Observed	Not observed	Observed	Not observed	Observed	Not observed	Observed	Not observed
**Sex (%)**											
	**Male**	35015 (47.6)	38515 (52.4)	39605 (53.9)	33928 (46.1)	14663 (19.9)	58853 (80.1)	27768 (37.8)	45747 (62.2)	16294 (22.2)	57233 (77.8)
	**Female**	5915 (12.7)	40655 (87.3)	16274 (34.9)	30307 (65.1)	3776 (8.1)	42798 (91.9)	7552 (16.2)	39021 (83.8)	2039 (4.4)	44531 (95.6)
**Age group (%)**											
	**35–44**	11849 (30.3)	27217 (69.7)	11412 (29.2)	27664 (70.8)	2668 (6.8)	36399 (93.2)	9243 (23.7)	29824 (76.3)	3779 (9.7%)	35287 (90.3)
	**45–54**	13855 (33.6)	27332 (66.4)	19142 (46.5)	22046 (53.5)	5872 (14.3)	35311 (85.7)	12120 (29.4)	29064 (70.6)	6290 (15.3)	34895 (84.7)
	**55–64**	12975 (37.2)	21881 (62.8)	21613 (62.0)	13245 (38.0)	8322 (23.9)	26526 (76.1)	12070 (34.6)	22777 (65.4)	6965 (20.0)	27890 (80.0)
	**65–75**	2251 (45.1)	2740 (54.9)	3712 (74.4)	1280 (25.6)	1577 (31.6)	3415 (68.4)	1887 (37.8)	3103 (62.2)	1299 (26.0)	3692 (74.0)
**Business Category (%)**											
	**(1) Agriculture**	340 (33.3)	682 (66.7)	504 (49.3)	518 (50.7)	181 (17.7)	841 (82.3)	313 (30.6)	709 (69.4)	157 (15.4)	865 (84.6)
	**(2) Mining**	182 (41.5)	257 (58.5)	261 (59.5)	178 (40.5)	85 (19.4)	354 (80.6)	157 (35.8)	282 (64.2)	90 (20.5)	349 (79.5)
	**(3) Utilities**	409 (47.2)	457 (52.8)	413 (47.6)	454 (52.4)	161 (18.6)	705 (81.4)	299 (34.5)	567 (65.5)	177 (20.4)	689 (79.6)
	**(4) Construction**	6365 (44.1)	8082 (55.9)	10813 (48.7)	11411 (51.3)	2800 (19.4)	11644 (80.6)	5244 (36.3)	9200 (63.7)	3031 (21.0)	11415 (79.0)

**Table 3:** Characteristics of Subjects (continued from Table 2).

	Waist circumference		Blood pressure		Blood glucose		Lipid		Metabolic Syndrome	
Abnormalities	Observed	Not observed	Observed	Not observed	Observed	Not observed	Observed	Not observed	Observed	Not observed
**(5) Manufacturing**	8157 (31.0)	18181 (69.0)	9368 (50.5)	9196 (49.5)	3550 (13.5)	22784 (86.5)	7211 (27.4)	19123 (72.6)	3466 (13.2)	22872 (86.8)
**(6) Wholesale Trade**	6222 (34.0)	12066 (66.0)	8054 (44.0)	10237 (56.0)	2668 (14.6)	15620 (85.4)	5231 (28.6)	13057 (71.4)	2645 (14.5)	15643 (85.5)
**(7) Transportation**	4520 (50.8)	4385 (49.2)	5306 (59.6)	3599 (40.4)	1968 (22.1)	6934 (77.9)	3457 (38.8)	5444 (61.2)	2290 (25.7)	6615 (74.3)
**(8) Information**	782 (45.0)	956 (55.0)	664 (38.2)	1074 (61.8)	220 (12.7)	1518 (87.3)	650 (37.4)	1088 (62.6)	296 (17.0)	1442 (83.0)
**(9) Finance**	418 (42.9)	557 (57.1)	437 (44.8)	538 (55.2)	165 (16.9)	810 (83.1)	339 (34.8)	636 (65.2)	180 (18.5)	795 (81.5)
**(10) Real Estate**	387 (32.8)	792 (67.2)	506 (42.9)	673 (57.1)	164 (13.9)	1015 (86.1)	348 (29.5)	831 (70.5)	168 (14.2)	1011 (85.8)
**(11) Professional Services**	1306 (38.8)	2063 (61.2)	1452 (43.1)	1918 (56.9)	528 (15.7)	2842 (84.3)	1143 (33.9)	2226 (66.1)	598 (17.8)	2771 (82.2)
**(12) Educational Services**	321 (33.0)	653 (67.0)	394 (40.5)	580 (59.5)	111 (11.4)	863 (88.6)	290 (29.8)	684 (70.2)	137 (14.1)	837 (85.9)
**(13) Health Care**	4142 (20.9)	15679 (79.1)	7701 (38.8)	12122 (61.2)	2275 (11.5)	17547 (88.5)	4248 (21.4)	15573 (78.6)	1734 (8.7)	18086 (91.3)
**(14) Arts**	1088 (28.6)	2711 (71.4)	1554 (40.9)	2245 (59.1)	504 (13.3)	3292 (86.7)	1009 (26.6)	2787 (73.4)	461 (12.1)	3338 (87.9)
**(15) Accommodation**	859 (29.6)	2044 (70.4)	1209 (41.6)	1695 (58.4)	421 (14.5)	2483 (85.5)	703 (24.2)	2201 (75.8)	350 (12.1)	2553 (87.9)
**(16) Cooperative Association**	1532 (39.2)	2372 (60.8)	1937 (49.6)	1967 (50.4)	727 (18.6)	3177 (81.4)	1373 (35.2)	2531 (64.8)	774 (19.8)	3130 (80.2)
**(17) Other Services**	3503 (36.6)	6080 (63.4)	4671 (48.7)	4914 (51.3)	1725 (18.0)	7857 (82.0)	2951 (30.8)	6632 (69.2)	1603 (16.7)	7979 (83.3)
**(18) Public Administration**	397 (25.6)	1153 (74.4)	635 (40.9)	916 (59.1)	186 (12.0)	1365 (88.0)	354 (22.8)	1197 (77.2)	176 (11.4)	1374 (88.6)

The SPR of abnormalities by business category for the male and female subjects are shown in Tables 4 and 5. Among the male subjects, significantly higher prevalences of metabolic syndrome were seen in the following four business categories: (4) Construction (1.04 [95% CI 1.00, 1.08]); (7) Transportation (1.21 [95% CI 1.16, 1.26]); (11) Professional Services (1.14 [95% CI 1.05, 1.24]); and (16) Cooperative Association (1.23 [95% CI 1.14, 1.33]). Males in the (7) Transportation industry showed higher prevalence in all abnormalities. In the (3) Utilities category, the males had a higher prevalence in excessive waist circumference only (1.16 [95% CI 1.05, 1.29]), and those in the (13) Health Care category had higher prevalences in hypertension (1.04 [95% CI 1.00, 1.08]) and glucose intolerance (1.07 [95% CI 1.01, 1.14]).

**Table 4:** Standardized Prevalence Ratio by Business Category Among Males.

	SPR (95% CI)				
	Excessive waist circumference	Hypertension	Glucose intolerance	Dyslipidemia	Metabolic syndrome
**(1) Agriculture**	0.91 (0.81, 1.02)	0.95 (0.86, 1.05)	1.00 (0.85, 1.18)	0.96 (0.84, 1.08)	0.89 (0.75, 1.05)
**(2) Mining**	0.95 (0.81, 1.10)	1.10 (0.97, 1.26)	0.96 (0.76, 1.20)	0.99 (0.84, 1.17)	0.99 (0.80, 1.23)
**(3) Utilities**	**1.16 (1.05, 1.29)**	0.97 (0.87, 1.08)	1.12 (0.95, 1.31)	1.03 (0.92, 1.17)	1.11 (0.95, 1.29)
**(4) Construction**	**1.03 (1.00, 1.06)**	1.01 (0.99, 1.03)	1.02 (0.98, 1.06)	**1.04 (1.01, 1.07)**	**1.04 (1.00, 1.08)**
**(5) Manufacturing**	*0*.*89 (0*.*87*, *0*.*91)*	1.00 (0.98, 1.02)	*0*.*86 (0*.*83*, *0*.*90)*	*0*.*90 (0*.*88*, *0*.*93)*	*0*.*85 (0*.*82*, *0*.*88)*
**(6) Wholesale Trade**	1.00 (0.97, 1.02)	*0*.*96 (0*.*93*, *0*.*98)*	1.02 (0.98, 1.06)	0.98 (0.95, 1.01)	0.98 (0.94, 1.02)
**(7) Transportation**	**1.14 (1.10, 1.17)**	**1.10 (1.07, 1.13)**	**1.09 (1.04, 1.14)**	**1.07 (1.04, 1.11)**	**1.21 (1.16, 1.26)**
**(8) Information**	**1.15 (1.07, 1.24)**	*0*.*89 (0*.*82*, *0*.*97)*	0.96 (0.83, 1.10)	**1.19 (1.10, 1.29)**	1.05 (0.93, 1.18)
**(9) Finance**	**1.13 (1.02, 1.25)**	0.96 (0.87, 1.07)	1.08 (0.91, 1.27)	1.08 (0.96, 1.21)	1.09 (0.93, 1.27)
**(10) Real Estate**	1.02 (0.91, 1.14)	0.97 (0.87, 1.07)	1.01 (0.85, 1.20)	1.06 (0.94, 1.20)	1.01 (0.86, 1.19)
**(11) Professional Services**	**1.11 (1.05, 1.18)**	0.96 (0.90, 1.01)	1.03 (0.94, 1.13)	**1.15 (1.08, 1.23)**	**1.14 (1.05, 1.24)**
**(12) Educational Services**	1.04 (0.92, 1.18)	0.95 (0.84, 1.08)	0.88 (0.71, 1.08)	**1.17 (1.03, 1.34)**	1.05 (0.87, 1.26)
**(13) Health Care**	*0*.*95 (0*.*91*, *0*.*99)*	**1.04 (1.00, 1.08)**	**1.07 (1.01, 1.14)**	0.98 (0.94, 1.03)	0.97 (0.92, 1.04)
**(14) Arts**	0.95 (0.89, 1.01)	*0*.*93 (0*.*87*, *0*.*99)*	1.00 (0.90, 1.11)	0.99 (0.92, 1.06)	0.94 (0.85, 1.04)
**(15) Accommodation**	0.95 (0.88, 1.02)	0.93 (0.86, 1.00)	1.05 (0.94, 1.18)	*0*.*90 (0*.*83*, *0*.*98)*	0.91 (0.81, 1.02)
**(16) Cooperative Association**	**1.12 (1.06, 1.18)**	1.04 (0.99, 1.10)	**1.17 (1.08, 1.27)**	**1.15 (1.09, 1.22)**	**1.23 (1.14, 1.33)**
**(17) Other Services**	0.98 (0.95, 1.02)	0.97 (0.94, 1.01)	1.02 (0.96, 1.07)	0.98 (0.94, 1.02)	0.96 (0.92, 1.02)
**(18) Public Administration**	1.05 (0.93, 1.19)	1.05 (0.94, 1.17)	0.90 (0.75, 1.09)	1.03 (0.89, 1.19)	1.04 (0.87, 1.23)

Italicized numbers indicate 95% CI of less than 1; Bold numbers indicate 95% CI of 1 or more.

**Table 5:** Standardized Prevalence Ratio by Business Category Among Females.

	SPR (95% CI)				
	Excessive waist circumference	Hypertension	Glucose intolerance	Dyslipidemia	Metabolic syndrome
**(1) Agriculture**	0.79 (0.56, 1.12)	0.98 (0.82, 1.17)	0.90 (0.61, 1.31)	0.92 (0.71, 1.20)	0.77 (0.43, 1.35)
**(2) Mining**	1.10 (0.54, 2.18)	1.02 (0.67, 1.55)	1.07 (0.44, 2.46)	1.26 (0.72, 2.17)	0.67 (0.12, 2.71)
**(3) Utilities**	1.09 (0.71, 1.64)	0.86 (0.65, 1.14)	0.56 (0.26, 1.16)	0.96 (0.65, 1.42)	0.92 (0.40, 1.98)
**(4) Construction**	0.99 (0.88, 1.12)	*0*.*90 (0*.*84*, *0*.*97)*	*0*.*84 (0*.*71*, *0*.*98)*	1.01 (0.91, 1.12)	0.97 (0.79, 1.19)
**(5) Manufacturing**	0.97 (0.91, 1.02)	**1.08 (1.05, 1.12)**	0.98 (0.91, 1.05)	0.96 (0.92, 1.01)	0.96 (0.87, 1.06)
**(6) Wholesale Trade**	*0*.*91 (0*.*85*, *0*.*98)*	1.02 (0.97, 1.06)	0.92 (0.84, 1.01)	*0*.*93 (0*.*88*, *0*.*99)*	*0*.*85 (0*.*75*, *0*.*97)*
**(7) Transportation**	1.09 (0.91, 1.30)	1.04 (0.93, 1.16)	0.94 (0.74, 1.19)	1.10 (0.94, 1.29)	0.90 (0.64, 1.25)
**(8) Information**	0.96 (0.69, 1.33)	*0*.*73 (0*.*58*, *0*.*93)*	*0*.*55 (0*.*30*, *0*.*99)*	0.79 (0.56, 1.12)	0.74 (0.36, 1.45)
**(9) Finance**	1.10 (0.78, 1.54)	0.86 (0.68, 1.09)	0.86 (0.51, 1.42)	1.30 (0.97, 1.74)	0.97 (0.49, 1.85)
**(10) Real Estate**	0.92 (0.71, 1.19)	*0*.*82 (0*.*70*, *0*.*97)*	*0*.*53 (0*.*34*, *0*.*81)*	0.94 (0.75, 1.18)	0.64 (0.37, 1.09)
**(11) Professional Services**	0.89 (0.75, 1.06)	*0*.*82 (0*.*74*, *0*.*92)*	0.86 (0.68, 1.09)	1.02 (0.88, 1.19)	0.75 (0.53, 1.05)
**(12) Educational Services**	1.16 (0.90, 1.49)	0.89 (0.75, 1.07)	0.65 (0.42, 1.02)	0.94 (0.73, 1.22)	0.96 (0.58, 1.57)
**(13) Health Care**	**1.07 (1.03, 1.12)**	1.00 (0.97, 1.03)	**1.12 (1.06, 1.19)**	**1.07 (1.03, 1.11)**	**1.17 (1.09, 1.26)**
**(14) Arts**	0.90 (0.78, 1.04)	0.99 (0.91, 1.07)	0.97 (0.82, 1.15)	0.99 (0.88, 1.12)	0.85 (0.66, 1.09)
**(15) Accommodation**	0.96 (0.83, 1.12)	0.96 (0.87, 1.05)	1.04 (0.87, 1.26)	*0*.*79 (0*.*68*, *0*.*92)*	0.85 (0.64, 1.13)
**(16) Cooperative Association**	1.10 (0.95, 1.27)	1.04 (0.96, 1.14)	1.11 (0.93, 1.32)	**1.22 (1.08, 1.37)**	**1.28 (1.02, 1.61)**
**(17) Other Services**	0.97 (0.88, 1.08)	0.95 (0.89, 1.01)	1.05 (0.93, 1.18)	0.94 (0.86, 1.02)	0.93 (0.78, 1.11)
**(18) Public Administration**	1.09 (0.92, 1.28)	0.84 (0.75, 0.95)	0.84 (0.66, 1.06)	0.96 (0.82, 1.13)	1.01 (0.75, 1.36)

Italicized numbers indicate 95% CI of less than 1; Bold numbers indicate 95% CI of 1 or more.

Among the female subjects, significantly higher prevalences of metabolic syndrome were observed in (13) Health Care (1.17 [95% CI 1.09, 1.26]) and (16) Cooperative Association (1.28 [95% CI 1.02, 1.61]). Females in the (13) Health Care industry showed higher prevalences in four abnormalities: excessive waist circumference (1.07 [95% CI 1.03, 1.12]), glucose intolerance (1.12 [95% CI 1.06, 1.19]), dyslipidemia (1.07 [95% CI 1.03, 1.11]), and metabolic syndrome (1.17 [95% CI 1.09, 1.26]). Moreover, in the female subjects, the (3) Utilities, (7) Transportation, and (11) Professional Services categories did not show significantly high prevalence of any of the abnormalities.

**Fig 1:**
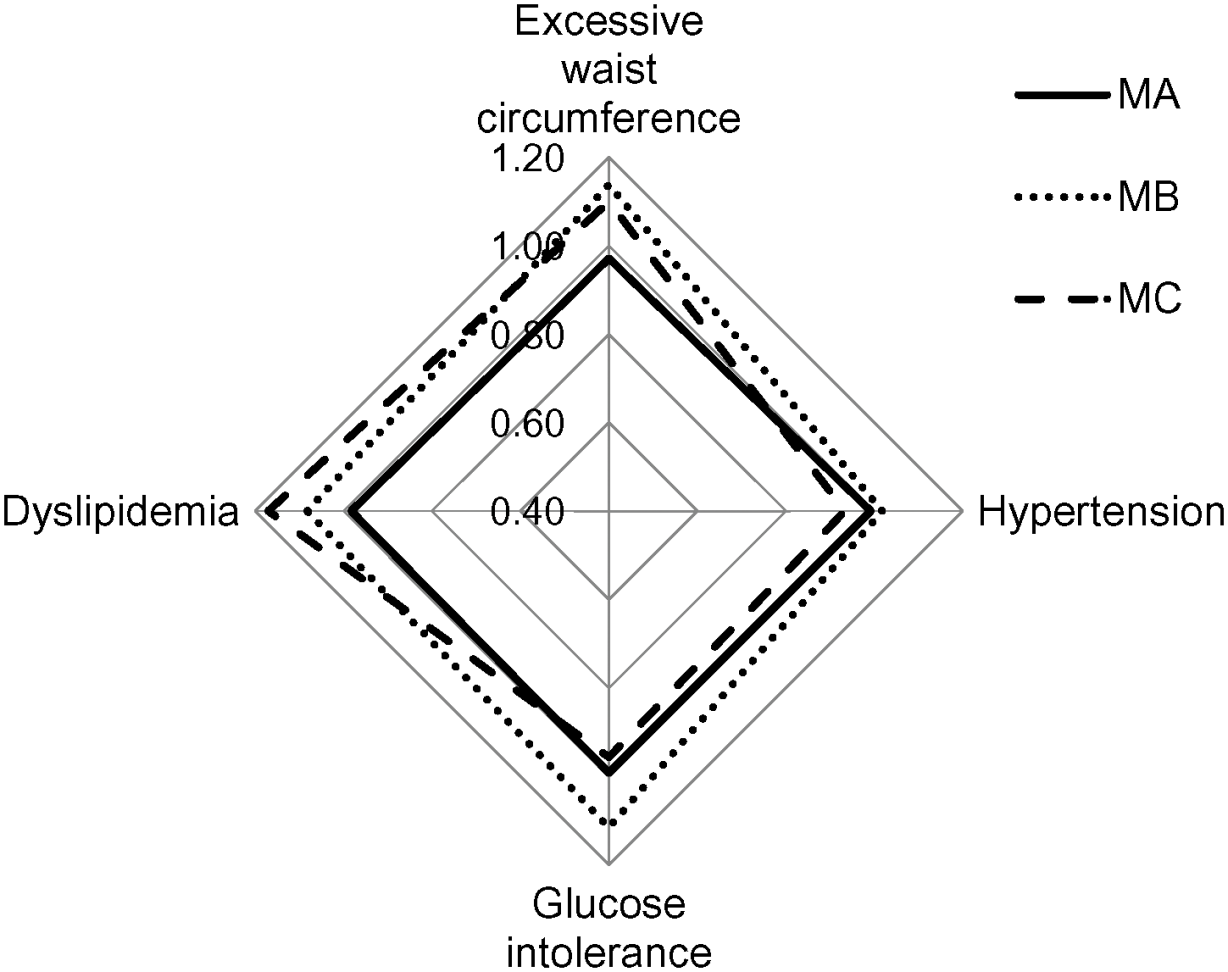
The clustering of business categories of males based on SPR. Cluster MA included (1) Agriculture, (2) Mining, (3) Utilities, (4) Construction, (8) Information, (10) Real Estate, (12) Educational Services, (13) Health Care, (15) Accommodation, (17) Other Services, and (18) Public Administration. Cluster MB included (5) Manufacturing, (7) Transportation, (9) Finance, and (16) Cooperative Association. Cluster MC included (6) Wholesale Trade, (11) Professional Services, and (14) Arts.

The results of the cluster analysis of males are shown in Fig 1. Cluster MA included (1) Agriculture, (2) Mining, (3) Utilities, (4) Construction, (8) Information, (10) Real Estate, (12) Educational Services, (13) Health Care, (15) Accommodation, (17) Other Services, and (18) Public Administration. Cluster MB included (5) Manufacturing, (7) Transportation, (9) Finance, and (16) Cooperative Association. Cluster MC included (6) Wholesale Trade, (11) Professional Services, and (14) Arts.

Among the male subjects, Cluster MA had an SPR lower than 1 for all components while Cluster MB had an SPR greater than 1 for all components. The SPRs of excessive waist circumference and glucose intolerance in Cluster MB were particularly higher than those in the other clusters (1.14 and 1.12, respectively). Cluster MC had an SPR lower than 1 for hypertension and glucose intolerance, and an SPR greater than 1 for excessive waist circumference and dyslipidemia. The SPR of dyslipidemia in Cluster MC was higher than that in other clusters (1.17).

**Fig 2:**
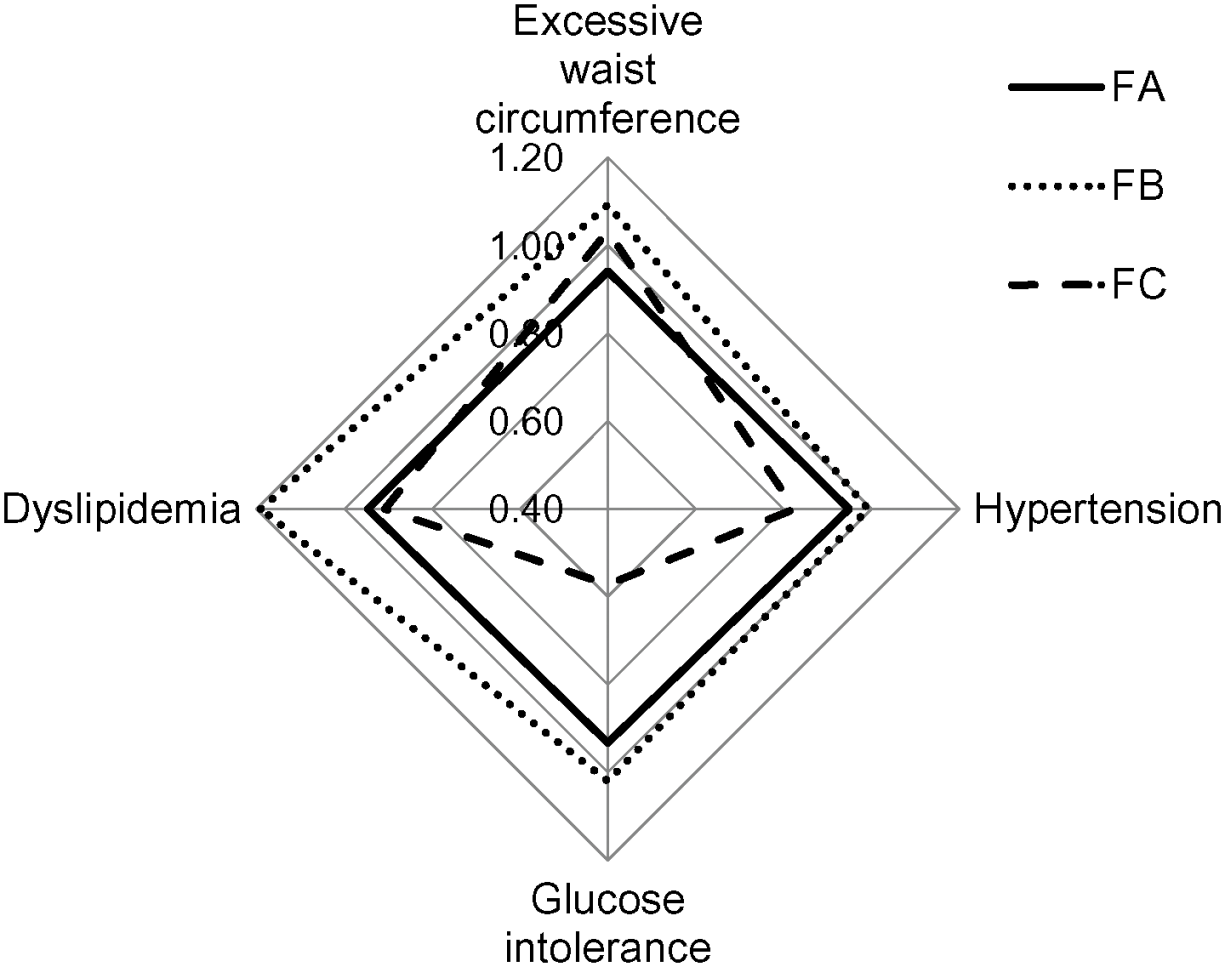
The clustering of business categories of females based on SPR. Cluster FA included (1) Agriculture, (3) Utilities, (4) Construction, (8) Information, (11) Professional Services, (12) Educational Services, (13) Health Care, (17) Other Services, and (18) Public Administration. Cluster FB included (2) Mining, (7) Transportation, (9) Finance, (15) Accommodation, and (16) Cooperative Association. Cluster FC included (5) Manufacturing, (6) Wholesale Trade, (10) Real Estate, and (14) Arts.

The results of the cluster analysis of the female subjects are shown in Fig 2. Cluster FA included (1) Agriculture, (3) Utilities, (4) Construction, (8) Information, (11) Professional Services, (12) Educational Services, (13) Health Care, (17) Other Services, and (18) Public Administration. Cluster FB included (2) Mining, (7) Transportation, (9) Finance, (15) Accommodation, and (16) Cooperative Association. Cluster FC included (5) Manufacturing, (6) Wholesale Trade, (10) Real Estate, and (14) Arts.

Among the female subjects, Cluster FA had an SPR lower than 1 for all components. Cluster FB had an SPR greater than 1 for excessive waist circumference, glucose intolerance and dyslipidemia (1.09, 1.02, 1.19, respectively), but not for hypertension. Cluster FC had an SPR greater than 1 for excessive waist circumference only (1.03); however, all other components had an SPR lower than 1, with glucose intolerance being particularly low (0.57).

## Discussion

In the current study, we investigated the prevalence of metabolic syndrome and its components by clustered business category, using big data. We found that metabolic syndrome was significantly prevalent among the male workers in the (4) Construction, (7) Transportation, (11) Professional Services, and (16) Cooperative Association industries, and among the female workers in the (13) Health Care and (16) Cooperative Association industries. Furthermore, the results of the cluster analysis indicated a cluster with a higher prevalence of metabolic syndrome components; for the male subjects, a cluster consisting of (5) Manufacturing, (7) Transportation, (9) Finance, (16) Cooperative Association, and for the female subjects, a cluster consisting of (2) Mining, (7) Transportation, (9) Finance, (15) Accommodation, and (16) Cooperative Association. We believe that the present study can provide an essential contribution to the understanding of the background of metabolic syndrome and its components.

Our study has also revealed that workers in (7) Transportation have a higher prevalence of glucose intolerance, whereas past studies indicated that such workers were at high risk of obesity, hypertension, dyslipidemia, and metabolic syndrome [10,20].

Among the female subjects, those in the (13) Health Care and (16) Cooperative Association categories had significantly higher SPR of metabolic syndrome. In the present study, we found that dyslipidemia was prevalent among (13) Health Care workers, whereas past studies have reported that such workers have high prevalence of obesity, diabetes, and metabolic syndrome [14,16,21]. The female (13) Health Care workers had significantly higher SPRs of all abnormalities except for hypertension, suggesting that they may be unhealthier than their male counterparts.

We also used hierarchical cluster analysis to group the business categories into three clusters according to the SPR. Among the male subjects, Cluster MA had a mean SPR of less than 1 for all components of metabolic syndrome. Thus, it is assumed that this cluster is a relatively healthier group than the other male clusters of the current study. In contrast, Cluster MB had a mean SPR of higher than 1 for all components of metabolic syndrome. This cluster is considered to be an aggregation of unhealthier business categories.

Among the female subjects, Cluster FA was considered to be a relatively healthier group as the mean SPR was less than 1 for all components. In contrast, Cluster FB had a higher mean SPR for all components except for hypertension. This cluster is considered to be the unhealthiest of the female clusters, and dyslipidemia is particularly prevalent here.

Furthermore, the past studies suggested that female workers in (11) Professional Services, (13) Health Care, and (18) Public Administration industries are at risk of obesity, hypertension, and glucose intolerance [11,16,21]. Our results, however, indicated that (2) Mining, (7) Transportation, (9) Finance, (15) Accommodation, and (16) Cooperative Association industries also had similar unhealthy features of metabolic syndrome components.

A limitation of this study was that the subjects’ precise occupations were unclear. As past studies have suggested, occupational factors affect the prevalence of metabolic syndrome and its components, which, for example, increases in workers whose work is sedentary [22]. Future studies should be designed to include such occupational factors as subcategories to business category.

In conclusion, we revealed the prevalence of metabolic syndrome and its components among Japanese workers by business categories and described the features of the prevalence. The business categories of (4) Construction, (7) Transportation, (11) Professional Services, and (16) Cooperative Association among the male subjects, as well as (13) Health Care and (16) Cooperative Association among the female subjects, were at a significantly high risk of metabolic syndrome. Furthermore, we were able to summarize the business categories into three clusters, based on the prevalence of the components of metabolic syndrome in both males and females. The Cluster MB in male and FB in female were identified as having a higher prevalence of metabolic syndrome components.

The findings of the present study show that the prevalence of metabolic syndrome or its components varies according to business category and gender, and must be taken into account when providing health guidance and support to patients with this disease.
